# Pneumothorax after a clinical breast fine-needle aspiration of a lump in a patient with Poland's syndrome

**DOI:** 10.1186/1477-7800-2-14

**Published:** 2005-08-19

**Authors:** M Salhab, W Al Sarakbi, N Perry, K Mokbel

**Affiliations:** 1 The Princess Grace Hospital, London, UK

**Keywords:** Pneumothorax, FNAC, breast cancer and Poland's syndrome.

## Abstract

We report the first case in the medical literature of a pneumothorax complicating fine needle aspiration cytology (FNAC) of a breast lump in a woman with a mild form of Poland's syndrome. The pneumothorax was treated conservatively. This is the first case of breast FNA-related pneumothorax seen in our clinical practice. We believe that the absence of pectoral muscles has increased the risk of this complication. We have also diagnosed an incidental screen-detected breast cancer affecting the ipsilateral breast in the same patient. We conclude that caution should be exercised when performing FNAC of breast lesions in patients with Poland's syndrome. The procedure should be preferably performed under image guidance in such patients in order to minimise the risk of this complication.

## Introduction

Fine needle aspiration cytology (FNAC) of the breast is a minimally invasive, safe, fast and cost effective technique which provides diagnostic information as to the nature of a breast lesion and can be used in appropriate settings to allow rapid management planning.

Pneumothorax occurring after FNAC of the breast is a recognized complication that has been reported in the literature. It was first described by Orr and Margarey in 1978 [1]. An Italian study of more than 200 000 FNAC procedures of the breast showed that pneumothorax occurred in 1 in 10000 cases (0.01%), however, the authors conceded that this figure might be underestimated due to unrecognized and asymptomatic cases of pneumothoraces [2,3].

Other studies have reported higher incidence of this complication; one in 417 by Kaufman [4] and one in 1000 by Gateley [5].

In this case we report a pneumothorax after FNAC of a breast lump in a woman with Poland's syndrome.

## Case report

**Figure 1:**
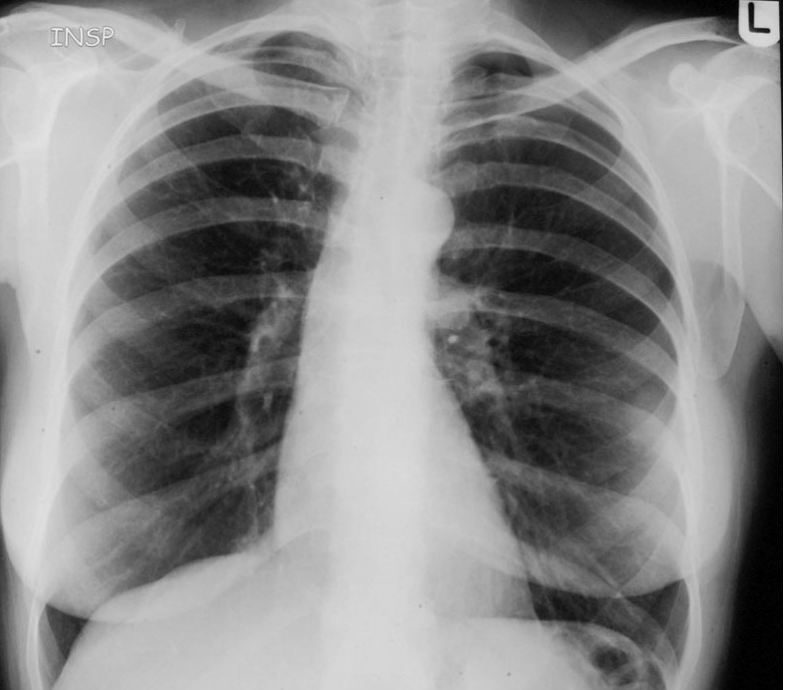
CXR in inspiration showing left sided small pneumothorax.

**Figure 2:**
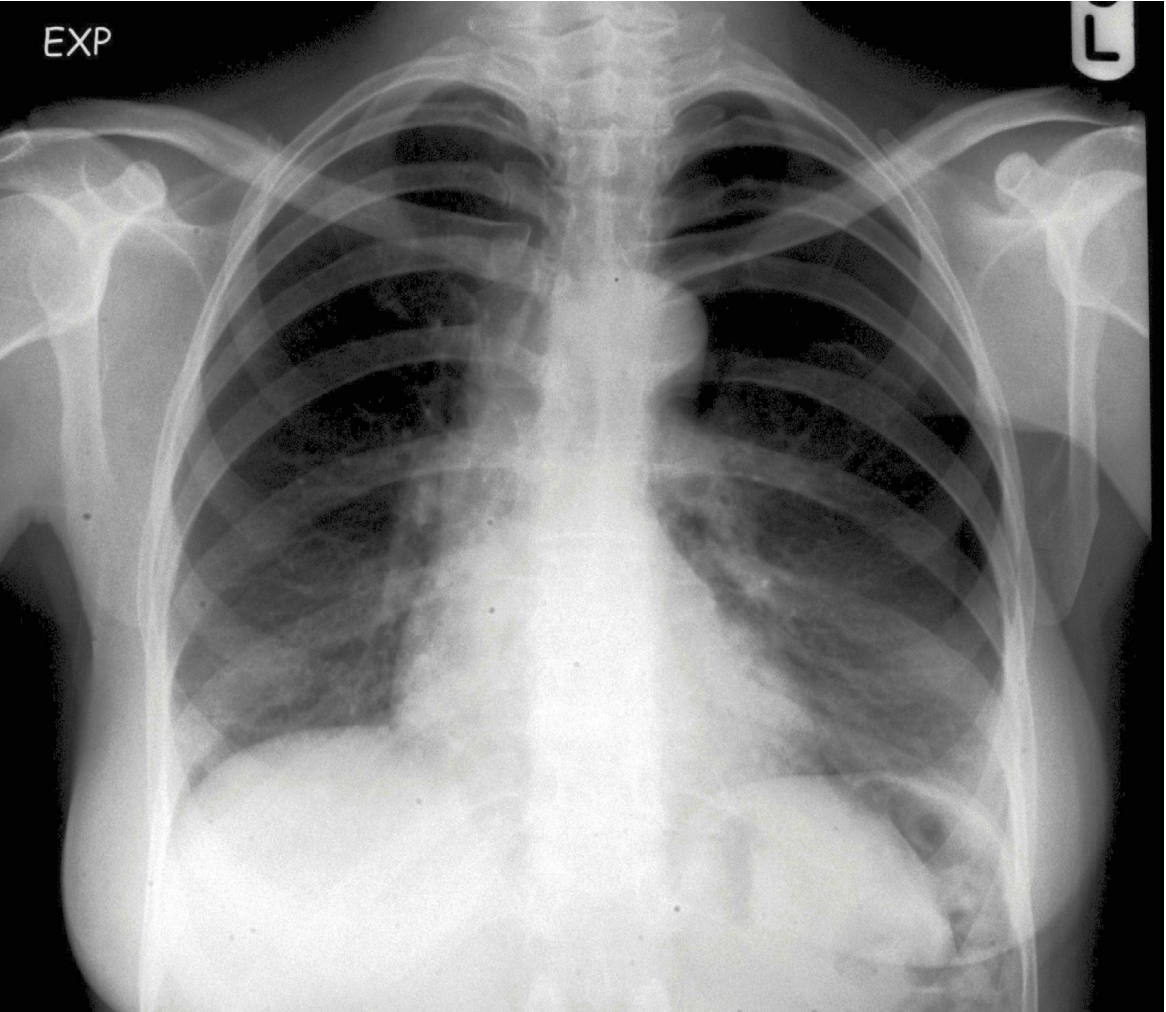
CXR in expiration showing obvious left sided pneumothorax.

A 52 years old female attended the breast clinic with a cystic mass superior to the left nipple. Clinical FNAC of this mass was performed (using a 23 gauge needle and 10 cc syringe) yielding 5 cc of straw-coloured fluid. Subsequently the patient had digital mammography. Thereafter, she complained of a difficulty in breathing and a subsequent chest X ray confirmed the presence of left-sided pneumothorax (Figures 1, 2) which treated with per-cutaneous aspiration. The patient was admitted overnight for observation and a follow up chest X-ray showed resolution of the pneumothorax. The post-procedure mammogram showed an area of architectural distortion and irregularity in the medial aspect of the left breast which was regarded as suspicious. Three days later, this was subjected to an ultrasound guided core biopsy of the lesion and histology showed severe atypical hyperplasia. Ultrasound guided excision of the lesion was performed and the final histology showed radial scar, a 7 mm grade I infiltrating ductal carcinoma associated with ductal carcinoma in situ (DCIS). The surgical margins were clear. The patient subsequently had a sentinel node biopsy using the dual localization technique under local anaesthesia.

**Figure 3:**
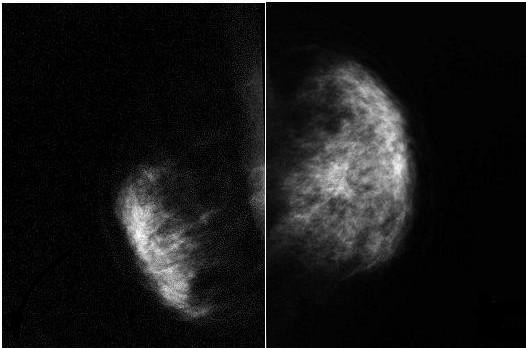
Digital mammogram (Mediolateral view) showing absence of the pectoralis major muscle and architectural distortion on the left side and normal right breast.

Digital mammography failed to adequately demonstrate the left pectoralis major muscle due to hypoplasia (Figure 3). Clinical examination confirmed the absence of the costo-sternal portion of the pectoralis major on the left side.

## Discussion

Pneumothorax as a complication of fine needle aspiration of the breast has been reported regularly in the medical literature over the last three Decades [1-10]. Pnemothorax is a relatively rare complication of breast FNAC partly due to the fact that the ribs form a much larger area of the chest wall surface than the intercostals muscles. Furthermore, the pectoral muscles provide additional protective structures. The reported incidence of this complication varies between 3 in 100 and 1 in 10000 [11]. Such an iatrogenic pneumothorax is usually treated conservatively without thoracostomy and drainage [12].

It is essential to perform the FNAC correctly in order to obtain a representative cytological sample from breast masses and avoid complications. To minimise the risk of developing pneumothorax after breast FNAC, it was suggested that the needle should be introduced parallel rather than perpendicular whenever possible to the chest wall therefore, avoiding overshooting of the needle tip which might pierce the intercostal muscles and injure the pleura and the underlying lung [13]. Patients should be encouraged to breathe normally while the procedure is being performed [4]. Furthermore it is advisable that patients with deep, centrally located and small or nonpalpable lesions have their FNAC under ultrasound-guidance. This is a safe and highly accurate procedure [14,15]. Pneumothorax after breast FNAC is seen more frequently in patients who have thin bodies and small sized breasts [3]. Furthermore, patients with deeply-located central lesions [4,8], or lesions in the tail of the breast [5] are also at higher risk for developing this complication.

In our case, the patient has an absence of the costo-sternal portion of the pectoralis major muscle on the same side where the breast FNA was performed, therefore the thickness of the chest wall behind the breast was much smaller than the contra-lateral side, therefore increasing the risk of inadvertent pneumothorax. Our patient has no obvious deformities. However, subsequent mammogram and clinical examination showed an absence of the pectoralis major; a case which is related to a rare condition called Poland's syndrome.

In 1841, Alfred Poland described an autopsy report of a 27 years old male with absence of the sternocostal portion of the pectoralis major, complete absence of pectoralis minor, hypoplasia of the serratus anterior and external oblique and defects in the middle phalanges [16]. In 1967 Baudinne et al coined the term Poland's syndrome [17]. This condition is very rare with a reported incidence of one in 20 000 – 32 000 live births and a 3:1 male predominance [18,19].

The clinical manifestations of Poland's syndrome vary, but typically it is characterized by a combination of hypoplasia or absence of the breast, nipple -areola complex and or the subcutaneous tissue [20], hypoplasia or absence of the costosternal portion of the pectoralis major muscle, serratus anterior or external oblique; absence of pectoralis minor muscle and absence of the costal cartilages of ribs 2,3,4 or 3,4,5. In addition, some patients have deformities of the hand and the upper limb [21].

Our patient has a minor manifestation of Poland's syndrome; the absence of the costo-sternal portion of the pectoralis major is a characteristic feature of this syndrome and is found in 100% of cases [19].

The association between Poland's syndrome and malignancy has been previously reported. Leukaemia, non-Hodgkin lymphoma, cervical cancer, leiomyocarcome, Wilms tumour and lung cancer have been reported in association with Poland's syndrome [22-27]. Furthermore, breast cancer has also been observed in females with this syndrome despite having mammary hypoplasia [28-34]. Interestingly, hypoplastic breasts may develop breast cancer similarly to normal breasts as observed by Havlik et al [28]. This observation raises important questions regarding a possible association between the two entities.

Pneumothorax in patients with Poland's syndrome has been previously reported but not in association with breast FNAC. Luh et al described two patients with Poland's syndrome anomalies presented with spontaneous pneumothorax. To the best of our knowledge our case is the first report of pneumothorax after FNAC of a breast lump in a woman with Poland's syndrome. We have also diagnosed an incidental screen-detected breast cancer affecting the ipsilateral breast.

Reconstructive surgery after mastectomy for breast cancer in patients with Poland's syndrome is challenging due to the absence or hypoplasia of the chest wall muscles thus necessitating the need to use myocutaneous flaps such as the latismus dorsi flap if implants are used. The TRAM, GAP and DEIP flaps represent alternative reconstructive techniques. Furthermore the subsequent use of radiotherapy in these patients carries a higher risk of lung complications due to the decreased protection by chest wall muscles.

In conclusion Poland's syndrome may be associated with an increased risk pneumothorax complicating FNAC of the breast. Therefore, this procedure should be carefully performed in such patients and preferably with image guidance in order to minimise the risk.
